# Weather Features Associated with Aircraft Icing Conditions: A Case Study

**DOI:** 10.1155/2014/279063

**Published:** 2014-02-20

**Authors:** Sergio Fernández-González, José Luis Sánchez, Estíbaliz Gascón, Laura López, Eduardo García-Ortega, Andrés Merino

**Affiliations:** Atmospheric Physics Group, IMA, University of León, 24071 León, Spain

## Abstract

In the context of aviation weather hazards, the study of aircraft icing is very important because of several accidents attributed to it over recent decades. On February 1, 2012, an unusual meteorological situation caused severe icing of a C-212-200, an aircraft used during winter 2011-2012 to study winter cloud systems in the Guadarrama Mountains of the central Iberian Peninsula. Observations in this case were from a MP-3000A microwave radiometric profiler, which acquired atmospheric temperature and humidity profiles continuously every 2.5 minutes. A Cloud Aerosol and Precipitation Spectrometer (CAPS) was also used to study cloud hydrometeors. Finally, ice nuclei concentration was measured in an isothermal cloud chamber, with the goal of calculating concentrations in the study area. Synoptic and mesoscale meteorological conditions were analysed using the Weather Research and Forecasting (WRF) model. It was demonstrated that topography influenced generation of a mesolow and gravity waves on the lee side of the orographic barrier, in the region where the aircraft experienced icing. Other factors such as moisture, wind direction, temperature, atmospheric stability, and wind shear were decisive in the appearance of icing. This study indicates that icing conditions may arise locally, even when the synoptic situation does not indicate any risk.

## 1. Introduction

The main consequences of aircraft icing are unusual loss of lift, such as a reduction in the rate of rise, an increase of friction, or rapid ice accumulation on windows, wings, or measurement instruments on the aircraft [1]. The analysis of aircraft icing is very important because of the numerous air crashes it has caused over recent decades [2].

Supercooled large drops (SLD) are drops of size greater than 50 *μ*m in a liquid state at temperatures below 0°C and constitute the principal source of aircraft icing. This is because such drops can freeze on aircraft structures that are unprotected or inadequately protected by anti-icing systems [3].

There are two possible mechanisms of SLD formation. The first is when frozen hydrometeors melt upon transiting regions with temperatures above freezing (often called “warm noses”) and reenter regions of subfreezing temperatures (resupercooling). The second mechanism is when liquid droplets form through a condensation process and grow into SLD by collision-coalescence processes, a cycle in which temperatures are less than 0°C [4, 5]. The first mechanism is often associated with warm frontal passage [6]. However, Strapp et al. [7] pointed out that approximately 75% of freezing precipitation events are a result of the second mechanism.

High humidity and updrafts are essential for the growth of supercooled water droplets, although there are other factors involved [8]. For efficient production of SLD by collision-coalescence processes, a mixing process is necessary [9]. Korolev and Isaac [10] claim that isobaric mixing produced by vertical air currents, which can produce supersaturation, may accelerate droplet growth to form SLD. This mechanism is favoured by an inversion layer near cloud top. Wind shear is another factor favouring SLD development because it induces mixing, which accelerates droplet growth and reduces the total number of drops [11].

At temperatures around −10°C, efficiency of the nucleation process is very low, because of weak activity of freezing nuclei at those temperatures [12]. Thus, this mechanism is not pronounced in clouds with tops at temperatures above −15°C. Rauber and Grant [13] indicated that supercooled liquid water (SLW) layers are common in orographic cloud systems with cloud tops at temperatures as low as −31°C. The cause of this phenomenon can be explained by imbalances between liquid water content (LWC) input by condensation and the nucleation rate, which is very slow [14]. To this it must be added that the freezing nuclei concentration is several orders of magnitude lower than that of condensation nuclei [15].

The average glaciation time in stratiform clouds is around 10 minutes [16]. Therefore, if the hydrometeor residence time is much smaller than the characteristic glaciation time, it is expected that virtually all hydrometeors will remain in the liquid phase. This is explained by Korolev and Isaac [10], who posit that SLD formed in updrafts have an average life of a few tenths of a second.

The processes discussed above are modified by terrain, making it necessary to perform a comprehensive mesoscale analysis. The importance of atmospheric flow modification caused by mountains depends on several parameters, such as mountain height and shape, atmospheric stability, wind speed and direction [17]. On the lee side of mountains, an area of weaker wind or eddy shedding can appear [18], and mountain waves can form [19]. Various works [20, 21] have shown the formation of mesolows on the lee side of several mountain ranges of the Iberian Peninsula, a result of a windflow perpendicular to the orographic barrier.

Reinking et al. [22] indicated that prefrontal flow is forced to ascend when it passes over an orographic barrier. Orographic lifting produces regions of LWC accumulation on the windward slope. After passing the orographic barrier, air descends abruptly and generates a cloudless area because of the Föehn effect. Subsequently, the flow rises suddenly, generating the characteristic gravity waves. Gravity waves form regions of short-lived but considerable amounts of LWC [23].

Numerical weather forecasting models are unable to forecast the concentration of SLD accurately, because commonly used parameterizations generally overestimate the amount of frozen water content and underestimate the concentration of supercooled liquid water [24].

Consequently, field campaigns using research aircraft to measure *in situ* supercooled liquid water and frozen water content are very important to improve the accuracy of numerical models. The vast majority of such field projects have been carried out in the USA and Canada [1, 25, 26], with some exceptions, as in Germany [27].

Under the TECOAGUA project, a series of flight plans has been designed to collect scientific data from in-cloud systems capable of producing rainfall during winter. These flights were executed by a C-212-200 aircraft, belonging to the National Institute for Aerospace Technology (INTA). One goal of this project was to fly in an icing environment to understand icing conditions that are not infrequent near Madrid-Barajas airport.

As noted by Baumgardner et al. [28], the Cloud Aerosol and Precipitation Spectrometer (CAPS) is suitable for measuring microphysical variables within clouds, so it was decided to install this instrument on the project aircraft with the aim of measuring SLD concentration. This probe is capable of measuring hydrometeor concentration and size (and distinguishing their phase), aerosols, LWC, temperature, relative humidity (RH), and vapour density, among other variables.

On February 1, 2012, the aircraft took off from the Torrejón de Ardoz military base (Madrid) and began collecting valid data at 12:57 (all times UTC). The aircraft flew north with the goal of collecting data on the north side of the Central System. Flying over the Lozoya Valley at altitude about 3500 msl, the aircraft penetrated a region with high SLD concentration and temperatures around −12°C, and LWC as much as 0.44 g/m^3^. This caused ice accumulation on the profile of the aircraft wings, forcing termination of the research flight.

The objective of the present study is to analyse the synoptic situation and mesoscale conditions during the day of this aircraft icing. To do this, weather conditions were measured by the available instrumentation, and the synoptic and mesoscale factors causing the icing were analyzed using the Weather Research and Forecasting (WRF) model.

## 2. Experimental Design and Methodology

### 2.1. Study Area

Icing of the C-212-200 aircraft on February 1, 2012, occurred while attempting to cross the Guadarrama Mountains. This mountain range is between the provinces of Segovia and Madrid, in central Spain. The orientation of ridges and valleys is predominantly southwest-northeast. The southwest end of the range is at 40°22′N, 4°18′W and its northeast end at 41°4′N, 3°44′W.

**Figure 1:**
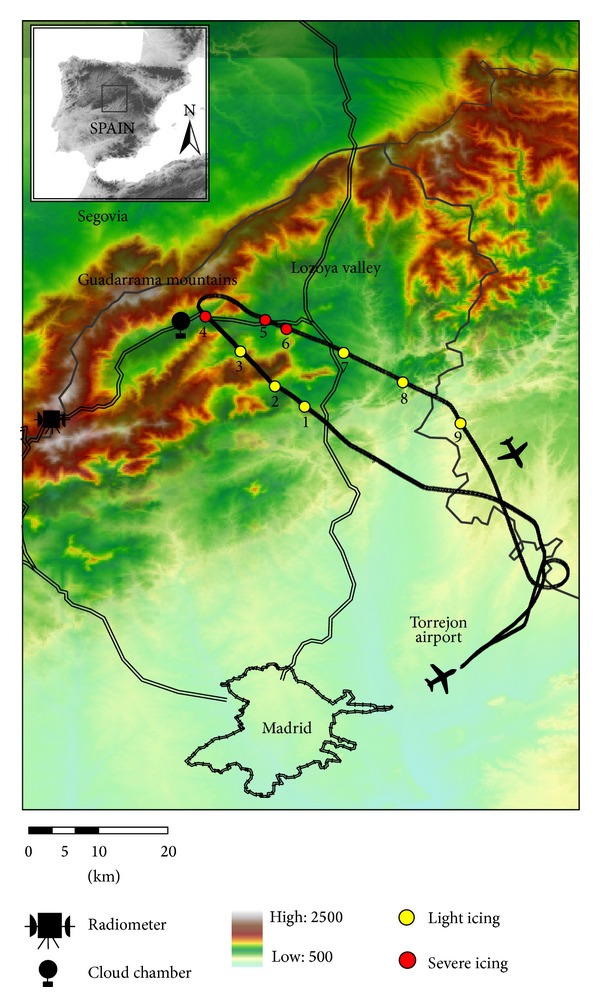
Map of Guadarrama Mountains, Madrid. Aircraft trajectory plus radiometer and cloud chamber locations have been superimposed.

Elevations in these mountains are well in excess of 2000 msl. The mountains are separated in the middle, with a higher range to the north and lower one to the south. The Lozoya Valley is between these two ranges. It is over this valley (Figure 1) where the aircraft traversed the region of high SLD concentration that caused rapid icing and flight termination.

### 2.2. Instrumentation

#### 2.2.1. MP-3000A Microwave Radiometric Profiler

For the 2011-12 winter field project, a hyperspectral, multichannel microwave radiometer (MMWR; MP-3000A) was installed at 1880 msl in the Guadarrama Mountains (location in Figure 1). The instrument continuously measured vertical profiles of temperature, humidity, LWC, and water vapour density (with temporal resolution approximately 2.5 minutes) to 10 km height. The MP-3000A was manufactured by Radiometrics, Boulder, Colorado, USA. Characteristics of this instrument and retrieved profiles are described in Sánchez et al. [29].

#### 2.2.2. CAPS

The C-212-200 aircraft carried the CAPS under the left wing during the field project. The CAPS consists of five probes. First is a Cloud and Aerosol Spectrometer (CAS), which measures aerosols and hydrometeor size distributions between 0.51 and 50 *μ*m. Second is a Cloud Imaging Probe Grayscale (CIP-GS), which can measure hydrometeors from 25 to 1550 *μ*m and represent 2D images of hydrometeors. The advantage of the grey scale is that it gives additional details on ice crystal characteristics and, more importantly, it better defines the depth of field and permits more accurate hydrometeor identification. Third is the LWD detector (LWC-Hotwire) probe, which estimates atmospheric LWC accurately between 0.01 and 3 g/m^3^. Finally, there was a sensor to measure airspeed and another to measure temperature and RH. A more detailed explanation of the CAPS is found in Baumgardner et al. [30].

#### 2.2.3. Isothermal Cloud Chamber

An isothermal cloud chamber was used to measure ice nuclei concentration of the air mass over the Guadarrama Mountains. This instrument was installed at ground level at Lozoya Reservoir (location in Figure 1) in the Lozoya Valley, where the aircraft experienced icing. This cloud chamber has a tank with volume 11 L. The ice nuclei concentration was measured at −23°C, because of the low concentrations recorded at warmer temperatures in previous Iberian Peninsula field projects. The procedure used in the campaign was described in Castro et al. [31]. This instrument is only capable of taking static measurements, so several measurements were taken during the study day to analyze the evolution of ice nuclei concentration.

## 3. Observation

The aircraft took off from Torrejón Airport at 12:57 on February 1, 2012. The aim of this flight was to investigate expected icing conditions from the approach of a cold front to the study area. Upon approach to the Guadarrama Mountains (13:10), the aircraft began to experience light icing. Icing conditions were worst after reaching the Lozoya Valley (13:15) and, consequently, the aircraft experienced severe icing and the pilot was forced to abort the mission and return to the airport. The aircraft continued to encounter severe icing until 13:19, when it exited the valley. Light icing occurred near the Guadarrama Mountains until 13:22, when the aircraft finally exited cloud. The aircraft landed at Torrejón at 13:31. The flight path is shown in Figure 1. The icing episode was recorded by the instrumentation as follows.

### 3.1. MP-3000A Radiometer

**Figure 2:**
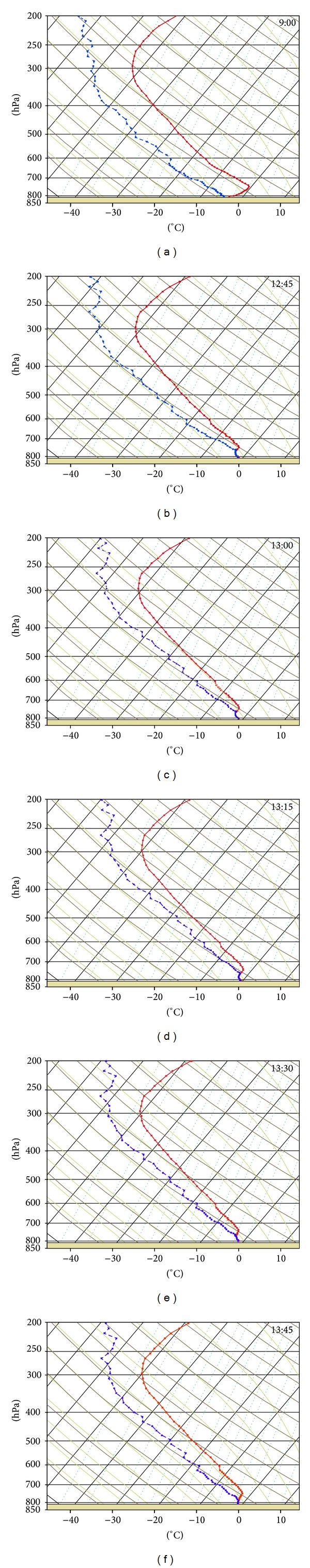
Skew-T/log-P diagram of radiometer data at 9:00, 12:45, 13:00, 13:15, 13:30, and 13:45 on February 1, 2012. Temperature (red lines); dew point (blue dashed lines).

Atmospheric stability was continuously monitored by the MP-3000A microwave radiometric profiler.  Figure 2 shows vertical profiles at different times. At 09:00 on February 1, it was observed that the nocturnal inversion had not dissipated at low levels. There was practically neutral stability from 720 hPa to 630 hPa, where there was a transition layer to greater stability at levels 600 hPa and above. There was neutral static stability (nearly unstable) between 750 and 600 hPa, above which was a strong stable layer.

At 12:45 when the aircraft neared the zone of interest, layers near the ground were saturated and the inversion had weakened considerably. Vertical profiles generally indicated an increasingly unstable atmosphere. Subsequently, the most unstable atmospheric layer developed from 750 hPa to 600 hPa. There was a progressive increase of moisture in this layer during preflight (12:00 to 13:00). There were clouds in this layer, and this was where the aircraft experienced icing (CAPS detected a region with high SLD concentration at 650 hPa). From 13:15 to 13:30, around which time the aircraft experienced severe icing, the temperature and dew point temperature curves between 750 and 600 hPa were close, indicating cloudiness. Subsequent profiles (13:45–14:00) indicated lesser instability and humidity, so we conclude that the aircraft crossed the Lozoya Valley during the most favourable conditions for aircraft icing all day.

Above the aforementioned unstable layer (just below 600 hPa) was a more stable region that persisted the entire day, representing a weak thermal inversion. This layer prevented updrafts in the Lozoya Valley from reaching higher levels and favoured formation of vertical shear. Bernstein [32] showed that stably stratified conditions are conducive to formation of regions of high SLD concentration. This would favour shear near cloud tops, which causes intense mixing and thereby efficient and rapid SLD formation [33].

The thermodynamic profile revealed the absence of a “warm layer,” indicating that SLD causing aircraft icing were formed by condensation followed by collision-coalescence, the entire process occurring at temperatures colder than 0°C. These profiles match “type A” described by Bernstein [32], in which the entire atmosphere has subfreezing temperatures, including the saturated layer.

The MMWR data permit continuous calculation of stability indices and determination of the presence or absence of convection. Although there are many indices that can be applied to this purpose in summer, there are few such for winter application. Most Unstable Convective Available Potential Energy (MUCAPE) has been widely used as a variation on CAPE in studies of winter convection [34–36]. This index represents the total potential energy available to the most unstable air parcel within the lowest 300 mb, while being lifted to its level of free convection. To obtain this index, CAPE must first be computed by lifting parcels from each level in the humidity and temperature profiles. Then, MUCAPE is taken as the greatest CAPE encountered, that is, the most unstable parcel.

**Figure 3:**
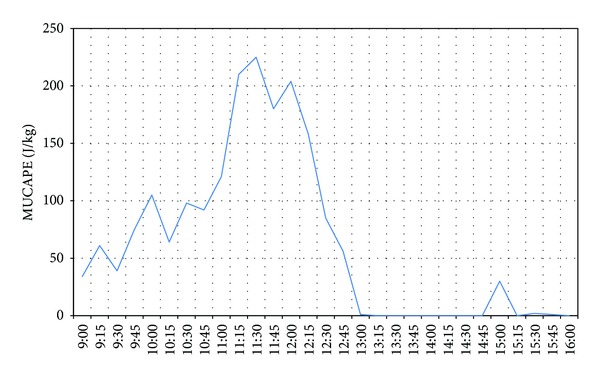
Evolution of MUCAPE index during February 1, 2013.

For continuous monitoring, the radiometer data were used. Intermediate values of MUCAPE were recorded in the hours before the flight (100–200 J/kg), but these values declined to 0 J/kg at 13:00 (Figure 3). These values are inadequate for convective development [37, 38].

### 3.2. CAPS

Hydrometeor size distributions from data collected by the CAPS during the flight of February 1 were analyzed in the region of aircraft icing. The gamma distribution function was chosen for these distributions, because it faithfully represents droplet size distributions within clouds. This function was defined by Ulbrich [39] and was selected because it better represents larger droplets. The function has three parameters dependent on time (and space in the Eulerian case): the concentration of particles, their average diameter, and spectrum width [40]. The parameters were estimated following the maximum likelihood method defined by Wilks [41]. The gamma distribution function has been used for representation of the size distribution of cloud hydrometeors [42]. The Lilliefors test [43] was used to ensure goodness of fit at the 0.05 level of significance.

**Figure 4:**
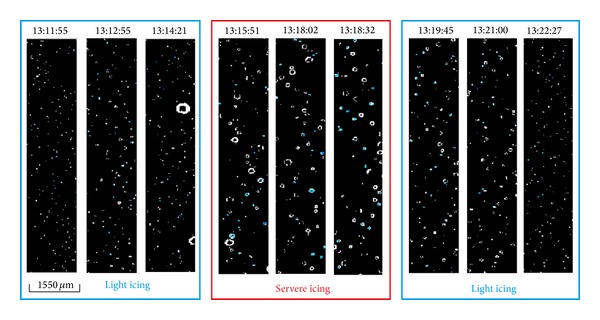
Images captured by CIP when aircraft experienced icing.

**Figure 5:**
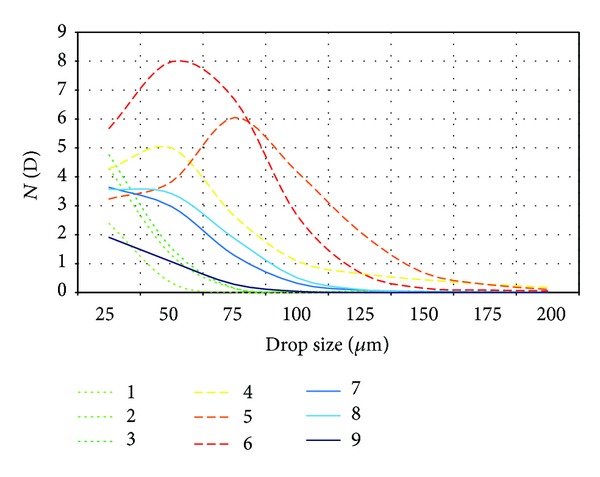
Gamma distribution curves for each of the nine times of Table 1 and Figure 4.

Average hydrometeor size distributions were calculated every 30 seconds. CAPS measurements in cloud are shown in Table 1. Images from the CIP (part of CAPS) for each period in Table 1 are shown in Figure 4. These nine measurements correspond to the highlighted circles along the trajectory in Figure 1, described previously in Section 2.1. Corresponding drop size distributions are shown in Figure 5, for which a gamma distribution function has been applied.

Initially, the predominant droplet size was <25 *μ*m. As the aircraft approached position 1, an increasingly large number of hydrometeors with size 25–50 *μ*m were measured. At that time, the aircraft experienced light icing but no loss of lift. This period corresponds with the three first times of Table 1 and first three images of Figure 4. Curves of the hydrometeor size distribution (curves 1, 2, and 3 in Figure 5) did not fit the gamma distribution.

Approaching position 3, the aircraft suddenly reached an area with a high concentration of droplets with size 50–100 *μ*m. This produced severe icing, forcing the pilot to turn around toward the south. He tried to climb out of cloud, but the SLD concentration at heights around 3800–3900 msl was even greater than 200 m below. There the aircraft encountered the worst icing conditions. This cloud had large quantities of LWC and SLD at temperatures around −12°C, freezing liquid drops as soon as they contacted the aircraft fuselage. The severe icing corresponds with the 4th, 5th, and 6th time steps of Table 1. The LWC increased to more than 0.2 g/m^3^, with peaks 0.44 g/m^3^. The table indicates the huge amounts of SLD and hydrometeors over 50 *μ*m. In the 4th, 5th, and 6th images of Figure 4, it is seen that SLD were larger than the small supercooled droplets in the other images. Figure 5 shows how the 4th, 5th, and 6th curves adjusted to the gamma distribution, unlike the remaining times. This is very important because it indicates that if the size distribution of supercooled liquid droplets follows the gamma distribution, we can expect moderate to severe icing; if it does not do so, light icing is the maximum expected.

After exiting the Lozoya Valley and the cloud with SLD toward the south, average drop size gradually decreased to predominant droplets smaller than 25 *μ*m, with light icing. This period corresponds with the 7th, 8th, and 9th times of Table 1, the images in Figure 4, and curves in Figure 5. The images captured by the CIP, droplet size distributions and values in Table 1 for times 7, 8, and 9 are similar to times 1, 2, and 3, except that the temperature was slightly cooler because the aircraft ascended to 3800–3900 msl.

Microphysical conditions observed over the Lozoya Valley by the aircraft during February 1 are consistent with those described by Ellrod and Bailey [44]. They stated that icing is linked with temperatures between 0 and −20°C, liquid- or mixed-phase clouds, volume median diameter greater than 30 *μ*m, LWC > 0.2 g/m^3^, weak updrafts to replenish supercooled liquid water, and clouds of considerable thickness. SLD can be very dangerous to aviation because they can accumulate beyond the capabilities of current deicing boots. This significantly reduces aircraft performance, which cannot be alleviated by ice protection devices such as pneumatic boots [45]. This occurred during the case study.

Cober et al. [1] described favourable environments for SLD development, in which supercooled liquid water droplets greater than 50 *μ*m form following melting and subsequent resupercooling or via condensation processes followed by collision-coalescence, which occurred in our case. Severe icing is formed by an updraft that provides sufficient water vapour to maintain SLD growth, while existing shear near the cloud top supports collision-coalescence processes responsible for its formation.

These results agree with those of Rauber and Tokay [14]. They asserted that when the concentration of freezing nuclei is low, cloud top temperature is relatively warm (above −20°C) with weak updrafts, and the likelihood of a LWC layer at cloud top is high. Based on data from scientific flights in field studies, Sand et al. [46] found that only 4% of icing reports were at temperatures below −20°C, with about 50% between −12°C and −8°C. Vidaurre and Hallet [47] noted that liquid-only clouds dominate at subfreezing temperatures close to 0°C, whereas ice-only clouds predominate below −20°C.

### 3.3. Isothermal Cloud Chamber Observations

**Figure 6:**
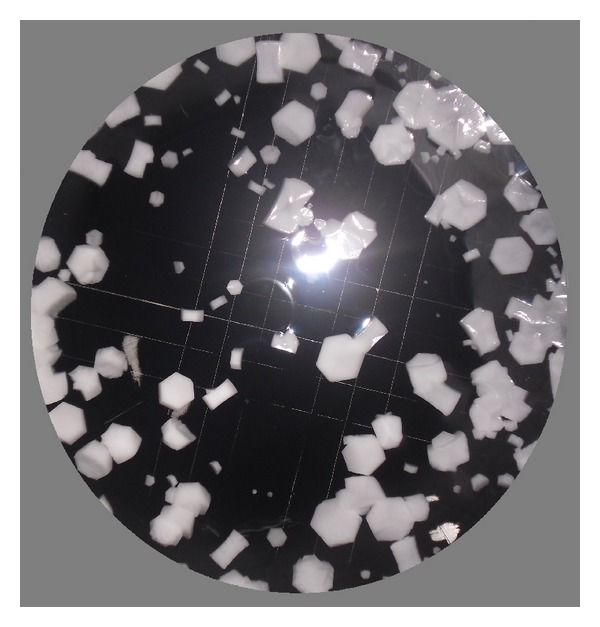
Measurement of ice nuclei from isothermal cloud chamber at 13:29.

Two measurements with the isothermal cloud chamber were taken on February 1. The first was at 9:58, resulting in a concentration of 24 IN/L (IN are ice nuclei). The second was around flight time at 13:29, showing a decrease to 16 IN/L (Figure 6). The thermal inversion during the morning, which disappeared after midday, may have been responsible for the high ice nuclei concentration of the first measurement. The breaking of the inversion layer at the surface allowed dispersion of IN to higher levels of the troposphere. The surface IN concentration at 13:29 was representative of that during the flight, because there was no inversion layer below 600 hPa.

These values are extremely low compared with those reported by other authors. After taking nearly 1000 measurements in the northwestern Iberian Peninsula, Castro et al. [31] obtained an average of 125 IN/L active at −23°C on days with maritime air masses. Our relatively sparse IN represents an obstacle to glaciation, which was a determining factor for the large numbers of SLD during the flight and near absence of hydrometeors in the solid phase.

At 600 hPa, near the cloud tops, the temperature was slightly warmer than −20°C. This temperature was sufficiently warm to prevent activation of most of the IN, hindering optimal glaciation.

## 4. Meteorological Analysis

WRF and other mesoscale models have been used for analysis of aircraft icing episodes [48, 49]. In this paper, weather conditions generating icing of the C-212-200 aircraft on February 1 were simulated by the WRF mesoscale model, version 3.1.1 [50]. Initial and boundary conditions were furnished by the National Centers for Environmental Prediction (NCEP) reanalysis, which has spatial resolution 1° [51].

Three nested domains were defined. D01 covers southwestern Europe, with 98 grids in both the eastwest and northsouth directions. This domain has spatial resolution 27 km and temporal resolution 3 hours and was used for the synoptic description.

Temporal resolution of domain D02 is 1 hour. It covers the entire Iberian Peninsula with 125 grid points in the eastwest direction and 107 northsouth points, with spatial resolution 9 km.

**Figure 7:**
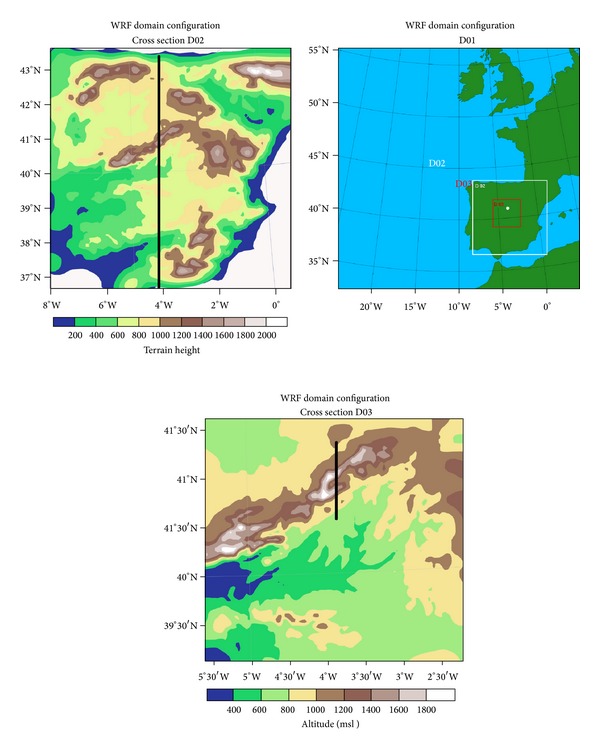
Nested domains used in WRF simulation. Axis from cross section from N to S is overlaid on D02 and D03.

To analyze in detail the mesoscale factors that influenced formation of icing conditions, domain D03 was used. This domain facilitates accurate representation of weather conditions in the study area, because it has spatial resolution 3 km and temporal resolution 1 hour.  Figure 7 shows the three domains. D02 and D03 were used in mesoscale analysis, D01 for synoptic analysis. The cross section axis is perpendicular to Guadarrama Mountains. Mesoscale models are commonly used in forecasting and evaluation of in-cloud icing conditions [52].

For parameterization of microphysical processes, the WRF New Thompson graupel scheme [53] was chosen, since it considers graupel and typical ice water concentrations in mountainous areas during winter. Further, we used the Noah land surface model [54] and Eta surface layer scheme defined by Janjic [55]. For longwave radiation, the Rapid Radiative Transfer Model [56] was used, along with the scheme of Dudhia [57] for shortwave radiation.

### 4.1. Synoptic Overview

**Figure 8:**
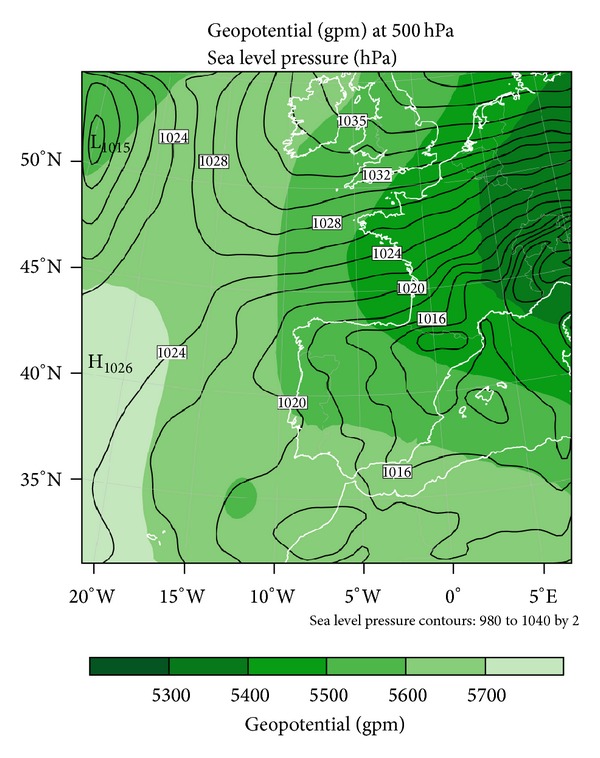
Sea level pressure and geopotential height at 500 hPa modeled by WRF for domain D01.

From domain D01, it was found that the synoptic situation in Europe was dominated by a powerful Siberian anticyclone (Figure 8), forcing a dry and very cold northeast wind into Central Europe. Northwest winds were predominant during the morning over the Iberian Peninsula.

**Figure 9:**
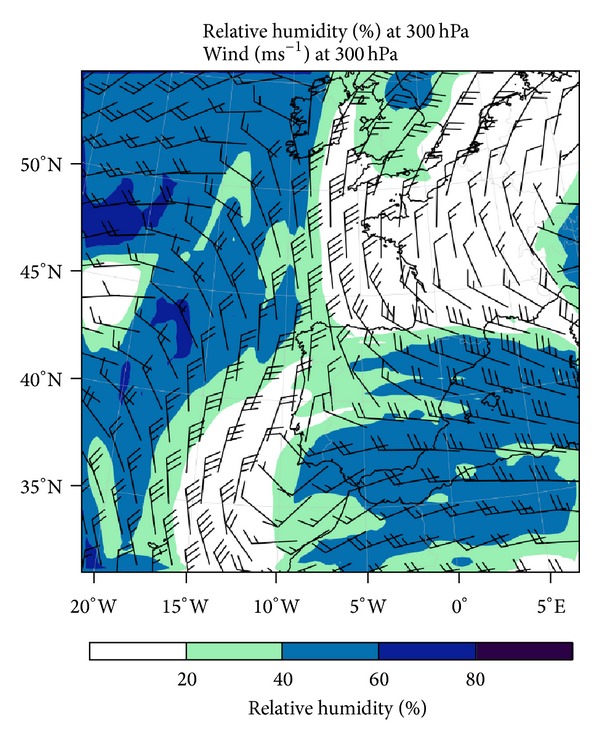
Relative humidity and wind at 300 hPa modeled by WRF in domain D01.

Figure 9 depicts RH and wind at 300 hPa. Progress of a dry intrusion induced the advection of moist air over the peninsula, in a manner similar to the pattern shown by Browning [58]. This dry intrusion is caused by a dynamic tropopause anomaly, which is derived from the jet stream. A weak cold front associated with the anomaly crossed the Iberian Peninsula from north to south during the study day. This synoptic pattern was associated with aircraft icing by Bernstein et al. [59]. The situation coincided with those reported by Bernstein et al. [11], who related the leading edges of arctic and cold fronts to in-flight icing episodes cited in pilot reports. This also fits the “Arctic Front” synoptic pattern described by Rauber et al. [60], who indicated that this pattern is the most common in freezing precipitation episodes.

There was another anomaly to the west of Lisbon at 12:00. A weakened branch thereof penetrated the Iberian Peninsula, at latitudes slightly south of Madrid. The two anomalies tended to associate, causing the accumulation and ascent of moist air in a strip between the two.

The formation of dynamic tropopause anomalies is connected with the position of the jet stream. Its location can be identified by the strong winds shown in Figure 9, depicted by wind barbs. The incursion of a subtropical anticyclone north of the Azores displaced the jet stream northward, while the powerful Siberian anticyclone pushed it southward, producing strong jet curvature. The jet stream was not clearly defined near the Iberian Peninsula but was separated into two branches. One was west of Portugal, and the other crossed the Pyrenees and moved toward the Mediterranean. The branches were associated with the two dynamic tropopause anomalies described above.

### 4.2. Mesoscale

The orographic forcing of the Guadarrama Mountains can be seen more clearly by increasing the model resolution. This forcing helped determine the generation and modification of factors that triggered mesoscale weather conditions supporting the icing. In the following, the causes of the icing are analyzed.

#### 4.2.1. Dynamic Tropopause Anomalies

**Figure 10:**
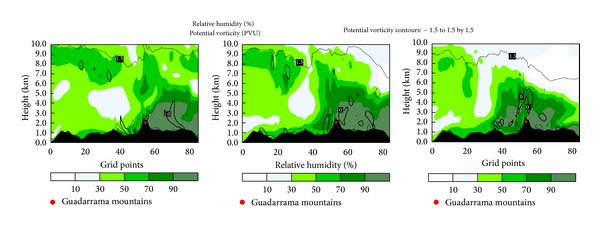
Cross section of relative humidity and potential vorticity in domain D02.

From domain D02 (see Figure 7) the vertical cross section of potential vorticity (PV) and RH is shown in Figure 10, in which the two dynamic tropopause anomalies are evident. A high PV region 7.5 km south of the Guadarrama Mountains corresponds to the anomaly observed to the southwest of Lisbon. This anomaly appears responsible for the midtroposphere dry air mass over the southern half of the Iberian Peninsula. A deeper anomaly was north of the Guadarrama Mountains, corresponding to the backside of the dry intrusion west of Italy. The rear of that anomaly moved southward, pushing an air mass with high humidity ahead of it. The convergence of the two anomalies accumulated moisture at the centre of the Iberian Peninsula. Substantial moisture is essential for SLD formation [2].

Nevertheless, updrafts over the Guadarrama Mountains cannot be attributed to the dynamic tropopause anomalies, because updrafts remained stationary on the lee side of the mountains throughout the day, while the anomalies moved to the south. Therefore, we conclude that the anomalies were not the main cause of the updraft that accumulated SLD over the Lozoya Valley. The backside of the anomaly at flight time remained north of the Iberian Peninsula; its effects were not observed in the mountains until after 18:00.

Associated with this anomaly was an advancing cold front. This front swept across the peninsula during the afternoon of February 1, resulting in ascent of the warm and wet air mass above a wedge of cold and dry air. Reinking et al. [22] stated that the ascent of a warm air mass over a cold one provides moisture and upwelling processes necessary for collision-coalescence growth.

#### 4.2.2. Mesolow

**Figure 11:**
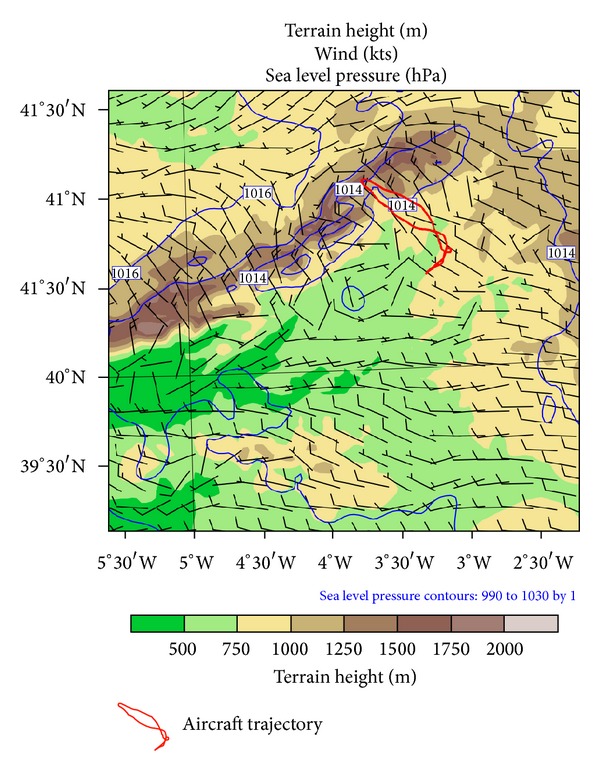
Terrain height, sea level pressure, and surface wind in domain D03. Aircraft trajectory has been superimposed.

By increasing model resolution in domain D03, it was observed that surface winds were perpendicular to the Guadarrama Mountains during the flight. This formed a mesolow on their lee side, caused by a phenomenon known as an orographic dipole. This is a mesoscale structure caused by flow perpendicular to a mountain barrier, forming anomalous positive pressure on the windward side and a depression on the lee side. Associated with the mesolow, surface wind had a cyclonic rotation on the lee side (Figure 11), which triggered wind convergence in the area of severe aircraft icing.

Orographic dipole formation is explained by separation of the boundary layer, a well-known phenomenon in fluid dynamics [61]. According to this theory, a steady stream encountering an obstacle generates a stagnation point and a pair of vortices; one is anticyclonic and upstream of the obstacle, and the other is cyclonic and downstream [62]. In such a 3D flow situation, additional effects should be considered, such as stratification, wave breaking, turbulence, and vertical wind shear [63].

#### 4.2.3. Updrafts and Gravity Waves

**Figure 12:**
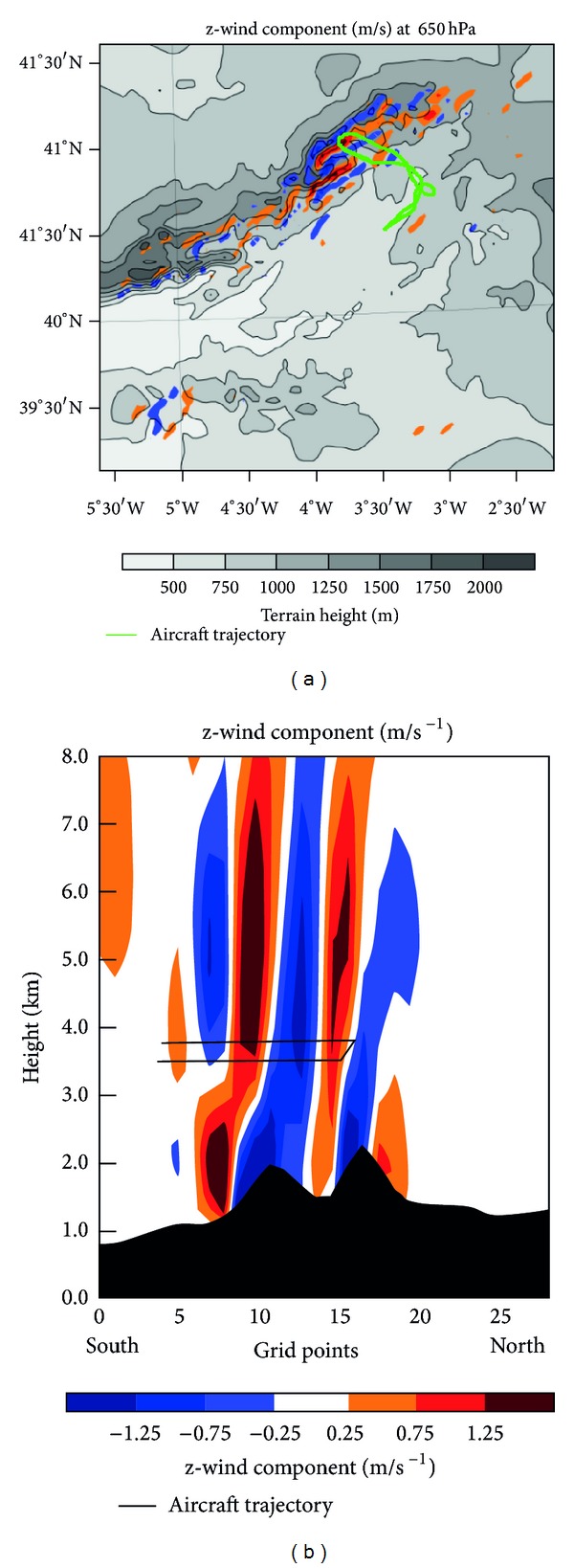
Vertical (z) wind component at 650 hPa (a) and on a cross section (b) of domain D03. Aircraft trajectory has been superimposed.

In the same area as the mesolow, an updraft appears in images produced by the WRF. This coincides with formation of the cloud band responsible for the aircraft icing. Geresdi et al. [64] indicated that icing regions often are associated with mesoscale uplift with vertical speeds around 5–20 cm s^−1^. In the vertical cross section of Figure 12, two updrafts are evident on the lee side of the Guadarrama Mountains. These updrafts are also seen in the vertical (z) wind component at 650 hPa, the level at which the aircraft experienced icing.

Coupled with the orographic dipole, mountain waves are common in regions of static stability (as shown by radiometer data) when the wind is perpendicular to the orographic barrier. This barrier offers resistance to passage of the flow. Unable to pass through the barrier, air tends to accumulate, resulting in loss of energy and wind speed reduction. This increases pressure on the windward side by wind convergence. Air passing through the mountains descends into the valley on the lee side and is then forced upward, generating mountain waves. Away from the mountains beyond a calm zone, the flow accelerates, producing divergence and a resulting pressure decrease [65]. The orographic dipole tends to strengthen updrafts generated by gravity waves.

Politovich [25] noted that orographic forcing may trigger convection embedded in stratiform clouds, which facilitates the ascent of SLD and their accumulation at cloud tops. Ikeda et al. [66] also asserted that strong flow perpendicular to a mountain barrier amplified vertical motions (up to 2 m/s) above local ridges, forming embedded convection.

Petersen et al. [67] indicated that if mountains are sufficiently high to block the prevailing flow, a mesolow or eddy shedding on the lee side is likely. In the WRF simulation, a mesolow was produced on the leeward side of the Guadarrama Mountains, owing to partial blockage of the perpendicular flow. The increase of PV caused by the mountains and accumulated at the mesolow reduced geopotential height at midtropospheric levels [68]. Additional PV may have come from approach of the cold front and dynamic tropopause anomaly discussed above. This caused a strong gradient of geopotential height to the lee of the mountains, as detected by an increase of wind speed and change in direction at midtroposphere, generating strong shear. This is consistent with Rauber [69], who claimed that gravity wave appearance in orographic cloud systems is usually associated with subsidence and strong shear at cloud tops. Here we should also note the cyclonic gyre of surface wind on the lee side of the Guadarrama Mountains, associated with the mesolow.

#### 4.2.4. Atmospheric Stability

We also analyzed Equivalent Potential Vorticity (EPV) to determine the existence of Conditional Symmetric Instability (CSI). After confirming that there was no negative EPV during the flight and no significant values of convective indices (analyzed using continuous radiometer measurements), the presence of convection was dismissed. We therefore conclude that the observed updrafts were caused by mountain waves, strengthened by the mesolow.

**Figure 13:**
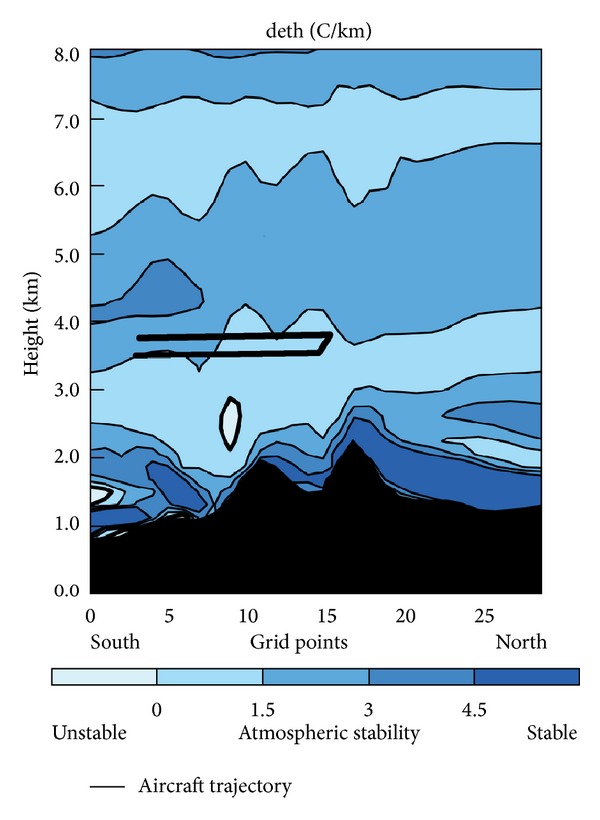
Cross section of deth in D03.

As seen in Figure 13 depicting differential equivalent potential temperature (deth), there was strong stability near the surface, with a thermal inversion in various areas of D03. Immediately above that inversion was a stable layer, which reached about 3000 msl. Most notable is the neutral stability layer located between 3 and 4 km, which allowed gravity wave development. Above this, there was another stable layer that prevented vertical development of the gravity waves above 4 km. Moreover, ripples caused by gravity waves can be discerned. Figure 13 portrays a small unstable region, coincident with the mesolow. The situation observed by the radiometer matches that modeled by the WRF, because if we represent deth in cross section, around 40.9°N (where the aircraft experienced icing) there was a neutral layer (almost unstable) from 2500 to 4000 msl. Immediately above 4000 msl, there was a stable layer.

Pobanz et al. [8] argued that shear above cloud top in a thermodynamically stable atmosphere can form a dynamically unstable layer and, thereby, turbulence and risk of Kelvin-Helmholtz waves as well as entrainment of subsaturated air and mixing, which favour SLD formation. Marwitz [70] had a similar theory. He affirmed that wind shear induced dynamical instability in gravity waves. However, the stable layer above 600 hPa blocked further ascent of air and thereby that of SLD. Therefore, the layer with greatest SLD accumulation formed immediately below this layer.

The nearly neutral static stability, together with weak updrafts associated with mountains, may be associated with production of SLW in this layer as pointed out by Pobanz et al. [8]. They claimed that a neutral or weakly unstable atmosphere promotes SLD formation. A static stability layer promotes generation of mountain waves. Orographic lifting is greater in a neutral atmosphere than in a stable one, fostering greater accumulation of SLD and LWC near cloud top. Furthermore, within a neutral stability region, cloud tops do not reach high altitudes. This is conducive to a low concentration of ice crystals, which facilitates the presence of SLD [71].

On the morning of February 1, 2012, there was a stable atmosphere with a strong inversion layer near the surface, as explained in the observation section. However, the conditions were increasingly unstable on the lee side of the Guadarrama Mountains, which were associated with formation of the mesolow. In addition, the cold front linked with the dynamic tropopause anomaly was approaching. As was the case in the present study, several authors have indicated that a transition from stable to more unstable conditions supports SLD formation [23, 72].

#### 4.2.5. Temperature

**Figure 14:**
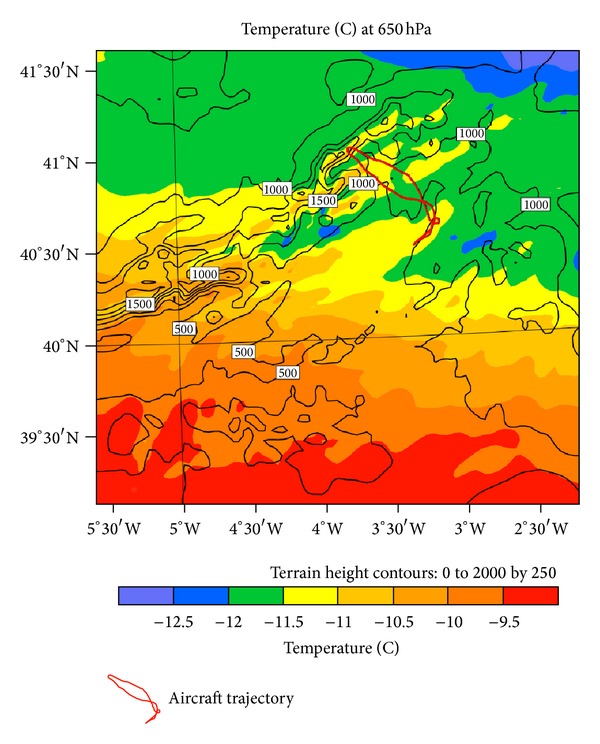
Temperature at 650 hPa in D03. Aircraft trajectory has been superimposed.

Figure 14 represents temperature at 650 hPa, near the aircraft icing altitude. Temperature there was about −12°C, an optimal value for such icing. This is because most freezing nuclei are not active and the nucleation process is therefore inefficient [12, 15].

The figure shows that over the Lozoya Valley where the first mountain wave was located, there was a band with lower temperatures than adjacent regions. A few miles south, there was another cold band collocated with a second mountain wave. Contiguous with these two regions, there were three bands with warmer temperatures than expected at this altitude; these correspond to subsidence regions produced by the mountain waves. A wave cloud occurs between the maximum and minimum vertical velocity, where temperatures are less than the undisturbed mean value [73].

#### 4.2.6. Liquid Water Content

**Figure 15:**
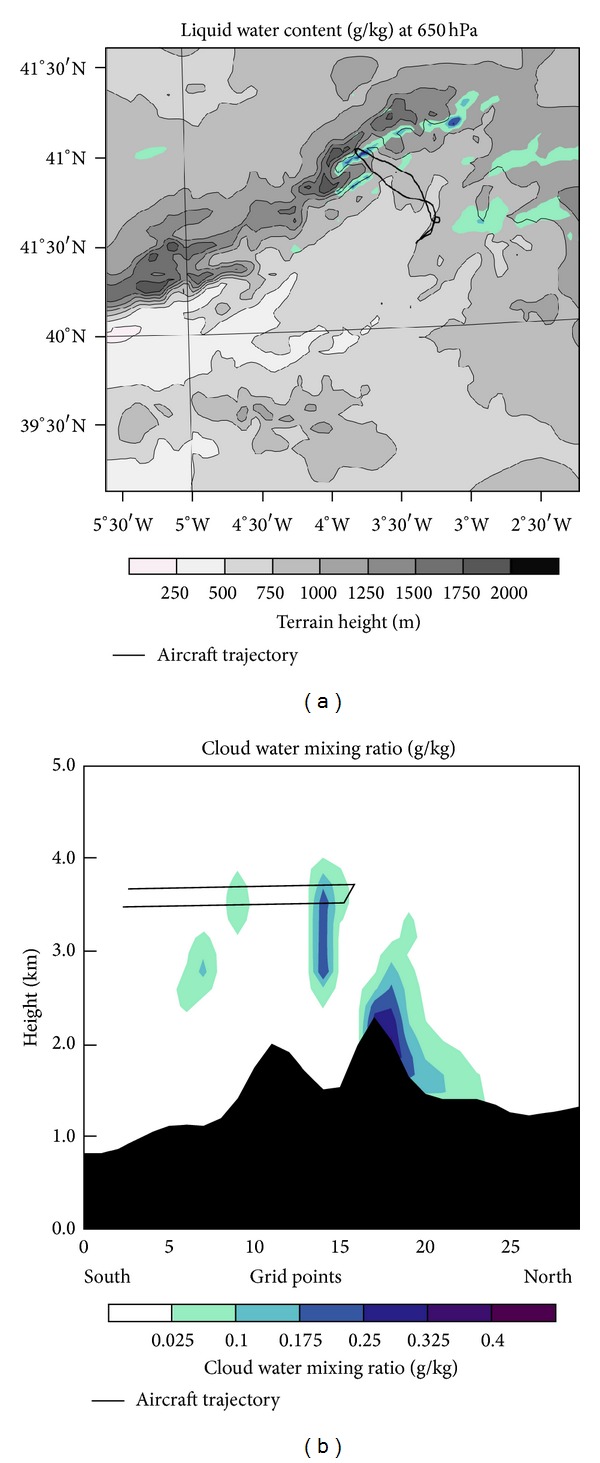
Liquid water content at 650 hPa (a) and on a cross section (b) of domain D03. Aircraft trajectory has been superimposed.

The cloud band over the Lozoya Valley shown in Figure 15 was responsible for the severe icing of the C-212-200. The aircraft was at 3500 msl (pressure 650 hPa), heading north. Arriving about 40.7°N, the aircraft entered cloud (mesolow area and second mountain wave) and experienced light to moderate icing (supercooled droplets smaller than 50 *μ*m and LWC about 0.1 g/m^3^) through 40.8°N. Upon reaching 40.9°N the aircraft reached the first mountain wave and a region of severe icing (supercooled large droplets greater than 50 *μ*m and LWC about 0.4 g/m^3^), forcing the mission abort.

Figure 15 explains why the aircraft experienced light icing in the first cloud, because LWC was low. Later, upon entering the zone of greater LWC, there was severe icing, coincident with the region of high SLD concentration. Just before the aircraft turnaround, a region with smaller droplets and low LWC was evident in images produced by the CIP-GS probe. This region corresponded with the northernmost mountain wave. Back to the south, the aircraft reentered the region of higher LWC and SLD, again enduring severe icing. The pilot ascended from 3500 to 3800 msl in an attempt to get above cloud but, as seen by the WRF output, cloud tops clearly exceeded this altitude.

During the return flight, the icing changed from moderate to light south of 40.8°N, and around 40.7°N the craft exited the cloud. There the pilot activated the anti-icing systems that detached the ice accumulated on the wing profile.

A cloudless band just downwind of the Guadarrama Mountains is evident in the WRF output. This band was caused by Föehn wind. These winds cause descending air and heating by compression on the lee side, dissipating the clouds. Subsequently, the air is forced to rise by mountain waves (with additional ascent caused by the mesolow in our case). This rising air cools by expansion, forming clouds associated with mountain waves after reaching the dew point level [22]. These authors showed that gravity waves produce significant amounts of supercooled LWC.

The region of aircraft icing had stratiform clouds with weak updrafts. Politovich [25] stated that when such clouds have maritime characteristics (as in this case, because the dominant air mass was maritime arctic), such as high humidity and very low IN concentration hindering ice crystal formation, they create an environment conducive to icing.

The updraft in the Guadarrama Mountains was the main cause of SLD accumulation in the region of aircraft icing, because it provided liquid water, mixing, and time for collision-coalescence processes. The updraft also allowed SLD accumulation, because it obstructed precipitation [74]. The updraft was generated by mountain waves and strengthened by the mesolow in the lee of the mountains.

After analyzing the D03 output of WRF, we concluded that the severe icing of the C-212-200 aircraft was caused by mountain waves. The aircraft first crossed the second (southernmost) mountain wave, experiencing light to moderate icing since the updraft there was less intense, and there was less LWC. However, the aircraft reached the northernmost mountain wave over the Lozoya Valley, experiencing severe icing caused by updrafts greater than 1 m/s and LWC in excess of 0.4 g/m^3^. In addition, the high SLD concentration caused ice accretion in areas unprotected by anti-icing systems, forcing flight termination. Stationary gravity waves are commonly dominated by cloud droplets smaller than 20 *μ*m but, in 2 m/s updrafts, drop sizes between 50 and 500 *μ*m can prevail, generating the greatest risk of aircraft icing [75].

## 5. Conclusions

In summary, severe icing of a C-212-200 aircraft during an approach to the Guadarrama Mountains on February 1, 2012 occurred as a result of several factors.

- Temperatures encountered by the aircraft at 3500 msl were optimal for icing, around −12°C. This fact, together with a low IN concentration (measured at the surface of Lozoya Valley by an isothermal cloud chamber during the flight), hindered the nucleation process. Furthermore, the radiometer registered a stable layer just below 600 hPa. This weak thermal inversion favoured formation of vertical shear increasing collision-coalescence process efficiency. LWC presence was detected by CAPS observation, which demonstrates that most of the hydrometeors in the gravity wave over the Lozoya Valley were liquid.
- In the WRF simulation, a dynamic tropopause anomaly approaching from north of the Iberian Peninsula pushed a warm and moist air mass ahead of it. Secondarily, another dynamic tropopause anomaly southwest of the peninsula supported moisture accumulation in the central peninsula. Airflow perpendicular to the Guadarrama Mountains during the hours before the flight formed a downwind mesolow, identified by an area of low pressure and a cyclonic gyre. This mesolow favoured convergence and updrafts in the region of aircraft icing. Together with the mesolow, mountain waves were the main cause of updrafts in the lee of the Guadarrama Mountains. These updrafts provided LWC, sufficient time for mixing, and SLD accumulation. A neutral atmosphere below 4 km altitude permitted the formation of gravity waves. Further, a stable layer above this altitude blocked development of these gravity waves, so a layer of high concentration of SLD and LWC appeared just beneath cloud top.

The combination of all these factors created an optimal environment for aircraft icing in a small region a few kilometres downwind of the Guadarrama Mountains, between 3500 and 4000 msl.

## Figures and Tables

**Table 1:** Variables measured by CAPS and gamma distribution parameters.

Time (UTC)	Latitude (°)	Longitude (°)	High (masl)	Temperature (K)	LWC (g/m^3^)	Number of particles by size ranges								*N*_*t*_	MVD (*μ*m)	Λ (cm^−1^)	*μ*	Gamma distribution fit
						0–25	25–50	50–75	75–100	100–125	125–150	150–175	175–200					
13:11:55	40.83	−3.64	3585	261.7	0.06	1082	84	2	1	1	0	0	0	1170	9	0.15	980	NO
13:12:55	40.87	−3.68	3600	261.5	0.12	1943	330	28	6	4	3	2	1	2317	11	0.18	1585	NO
13:14:21	40.92	−3.73	3570	261.6	0.16	2157	392	29	7	6	6	6	6	2609	65	0.19	1732	NO
13:15:51	40.95	−3.77	3589	261.6	0.21	1941	1284	513	179	98	63	40	24	4142	180	0.52	988	YES
13:18:02	40.95	−3.69	3894	260.3	0.28	1467	985	1174	689	315	99	39	15	4783	526	1.12	532	YES
13:18:32	40.93	−3.67	3895	259.8	0.35	2573	2051	1296	440	104	22	12	8	6506	302	0.62	1302	YES
13:19:45	40.90	−3.59	3922	259.5	0.11	1649	763	249	53	13	5	3	1	2736	34	0.34	1014	NO
13:21:00	40.87	−3.52	3897	259.6	0.11	1619	878	363	84	16	3	1	1	2965	39	0.40	937	NO
13:22:27	40.82	−3.44	3891	261.3	0.02	865	273	55	7	0	0	0	0	1200	5	0.24	614	NO
